# Supplementary material for: EnzML: multi-label prediction of enzyme classes using InterPro signatures
Source: BMC Bioinformatics. 2012 Apr 25;13:61. doi: 10.1186/1471-2105-13-61 (PMC3483700; doi:10.1186/1471-2105-13-61)
Supplement: Addtional file 5 — The Java code to format the data files, evaluate and predict. The file enzml_java_code.tar.gz contains the Java code used to format database data to ARFF and XML formats, to execute cross and train-test (jackknife) evaluations and to record evaluation results to database. More information is included in the readme.txt file and the Javadoc files. The code can be used with a MySQL database. To use a different database software, other JDBC drivers might be required. [file 1471-2105-13-61-S5.gz › java_code/enzml2011/doc/uk/ac/ed/inf/csb/taweka/DataSetWriter.html]

DataSetWriter


---


|  |  |  |
| --- | --- | --- |
| **Overview** \| **Package** \| **Class** \| **Use** \| **Tree** \| **Deprecated** \| **Index** \| **Help** | |  |
| **PREV CLASS**   **NEXT CLASS** | **FRAMES**    **NO FRAMES**     **All Classes** |  |
| SUMMARY: NESTED \| FIELD \| CONSTR \| METHOD | DETAIL: FIELD \| CONSTR \| METHOD |  |


---


## uk.ac.ed.inf.csb.taweka Class DataSetWriter

```
java.lang.Object
  uk.ac.ed.inf.csb.taweka.DataSetWriter
```

**Direct Known Subclasses:**: MulanDataSetWriter

---

``` public class DataSetWriter extends java.lang.Object ```

DataSet generates a collection of Weka instances (rows of the ARFF file),
their attributes (column names) and their attribute values (cell values) The
last attribute is the class attribute.

**Version:**
:   20 Feb 2008

**Author:**
:   Luna De Ferrari luna.deferrari-at-ed.ac.uk

---

| **Field Summary** | |
| --- | --- |
| `static java.lang.String` | `DUMMY_VALUE` |


**Constructor Summary**

`DataSetWriter(ArffGenerator generator,
uk.ac.ed.inf.csb.utils.OneToManyMap instanceAttributeMap,
uk.ac.ed.inf.csb.utils.OneToManyMap instanceClassMap)`


| **Method Summary** | |
| --- | --- |
| `void` | `generateDataSet()`             Create a set of instances and fill them with data |
| `ArffProperties` | `getArffProperties()` |
| `AttributeFactory` | `getAttributeFactory()` |
| `java.util.ArrayList<weka.core.Attribute>` | `getAttributes()` |
| `java.lang.String` | `getClassName()` |
| `java.lang.String` | `getDataSetName()` |
| `uk.ac.ed.inf.csb.utils.OneToManyMap` | `getInstanceAttributeData()` |
| `uk.ac.ed.inf.csb.utils.OneToManyMap` | `getInstanceClassData()`             public Instances getDataSet() { return m\_instances; } |
| `weka.core.Instances` | `getInstances()` |
| `java.lang.String` | `getLog()` |
| `java.util.Properties` | `getProperties()` |
| `void` | `writeInstancesToFile()` |

**Methods inherited from class java.lang.Object**

`equals, getClass, hashCode, notify, notifyAll, toString, wait, wait, wait`

**Field Detail**

### DUMMY\_VALUE

```
public static final java.lang.String DUMMY_VALUE
```

**See Also:**: Constant Field Values


**Constructor Detail**

### DataSetWriter

```
public DataSetWriter(ArffGenerator generator,
                     uk.ac.ed.inf.csb.utils.OneToManyMap instanceAttributeMap,
                     uk.ac.ed.inf.csb.utils.OneToManyMap instanceClassMap)
```

**Parameters:**: `generator` - a generator object to write this dataset to file: `instanceAttributeMap` - instance-attribute value entries: `instanceClassMap` - instance-class value entries


**Method Detail**

### generateDataSet

```
public void generateDataSet()
```

:   Create a set of instances and fill them with data

---


### getArffProperties

```
public ArffProperties getArffProperties()
```

---


### getAttributeFactory

```
public AttributeFactory getAttributeFactory()
```

---


### getAttributes

```
public java.util.ArrayList<weka.core.Attribute> getAttributes()
```

---


### getClassName

```
public java.lang.String getClassName()
```

---


### getDataSetName

```
public java.lang.String getDataSetName()
```

---


### getInstanceAttributeData

```
public uk.ac.ed.inf.csb.utils.OneToManyMap getInstanceAttributeData()
```

---


### getInstanceClassData

```
public uk.ac.ed.inf.csb.utils.OneToManyMap getInstanceClassData()
```

:   public Instances getDataSet() { return m\_instances; }

---


### getInstances

```
public weka.core.Instances getInstances()
```

---


### getLog

```
public java.lang.String getLog()
```

---


### getProperties

```
public java.util.Properties getProperties()
```

---


### writeInstancesToFile

```
public void writeInstancesToFile()
```


---


|  |  |  |
| --- | --- | --- |
| **Overview** \| **Package** \| **Class** \| **Use** \| **Tree** \| **Deprecated** \| **Index** \| **Help** | |  |
| **PREV CLASS**   **NEXT CLASS** | **FRAMES**    **NO FRAMES**     **All Classes** |  |
| SUMMARY: NESTED \| FIELD \| CONSTR \| METHOD | DETAIL: FIELD \| CONSTR \| METHOD |  |


---
